# Effect of nurse-led interventions on dyspnea severity and psychological well-being among patients with chronic obstructive pulmonary disease

**DOI:** 10.6026/973206300212733

**Published:** 2025-08-31

**Authors:** Malathi Dhakshnamoorthy, Vinod Kumar Viswanathan, Shankar Shanmugam Rajendran, Ananthi Duraikannu, Anitha Parasuraman, Gnana Malar Periyakaruppan, Jebamalar Rajan

**Affiliations:** 1 Department of Medical Surgical Nursing, College of Nursing, Madras Medical College, The TNMGR University, Chennai, Tamil Nadu, India; 2 Department of Respiratory Medicine, Institute of Respiratory medicine, MMC & RGGGH, The TNMGR University Chennai, Tamil Nadu, India; 3 Department of Child Health Nursing, College of Nursing, Madras Medical College, The TNMGR University, Chennai, Tamil Nadu, India; 4 Department of Community Health Nursing, College of Nursing, Madras Medical College, The TNMGR University, Chennai, Tamil Nadu, India

**Keywords:** COPD, Dyspnoea, Nurse-led intervention, psychological well-being, non-pharmacological therapy

## Abstract

The impact of nurse-led interventions on dyspnoea and psychological well-being in COPD patients is of interest. Hence, 60 stable COPD patients were divided into experimental and control groups. The experimental group received a comprehensive intervention, including breathing exercises, physical activity, dietary counselling, and psychological support, leading to significant improvements in dyspnoea and psychological well-being. In contrast, the control group showed no significant changes. Thus, we show the value of non-pharmacological nurse-led interventions in managing COPD and improving patient outcomes.

## Background:

Chronic Obstructive Pulmonary Disease (COPD) is a predominant cause of illness and death globally. In 2021, 99.7 million prevalent and 7.4 million incident COPD cases were recorded globally among adults ≥70 years. Deaths reached 2.9 million. While age-standardized rates declined, absolute case numbers increased by >150% since 1990 [1]. In India, the prevalence of COPD is considerable, with studies showing a rate between 4% and 10% among persons aged over 35 years [2]. Dyspnoea, or shortness of breath, is a defining symptom of COPD, resulting in diminished physical activity, muscular deconditioning, and psychological discomfort. Anxiety and depression are common among COPD patients, with studies indicating anxiety prevalence between 10% and 55% and depression prevalence between 10% and 42% [3]. These psychological comorbidities intensify the disease load and adversely affect quality of life. Non-pharmacological interventions, particularly nurse-led programs, have shown promise in managing COPD symptoms and improving psychological well-being. Breathing exercises such as pursed-lip breathing (PLB) and diaphragmatic breathing (DB) are effective techniques taught by nurses to alleviate dyspnea. PLB has been associated with a 19% improvement in oxygen saturation and a 21% reduction in respiratory rate during exercise [4]. DB has demonstrated a 28% increase in tidal volume and a 12% decrease in dyspnea scores [5]. Physical activity is another critical component of COPD management. Pulmonary rehabilitation programs, which often include nurse-led exercise training, have been shown to improve exercise capacity by 13% to 24% and reduce hospital admissions by 36% [6]. Regular physical activity also contributes to better psychological outcomes, with studies reporting a 20% reduction in anxiety and a 25% reduction in depression scores following structured exercise programs [7]. Dietary counselling provided by nurses plays a vital role in managing COPD. Malnutrition is prevalent in 25% to 40% of COPD patients and is associated with increased mortality. Nutritional interventions have led to a 1.5 kg increase in body weight and a 12% improvement in respiratory muscle strength [8]. Relaxation techniques, including progressive muscle relaxation and meditation, have been effective in reducing psychological distress in COPD patients. These interventions have resulted in a 23% decrease in anxiety levels and a 19% improvement in sleep quality [9]. Education on self-management and proper use of medical devices is another area where nurse-led interventions have made a significant impact. Patients receiving structured education demonstrated a 22% improvement in medication adherence and a 30% reduction in emergency department visits [10]. Therefore, it is of interest to assess the effectiveness of nurse-led interventions on dyspnea severity and psychological wellbeing in patients with Chronic Obstructive Pulmonary Disease.

## Materials and Methods:

This study employed a quantitative, quasi-experimental design to assess the efficacy of a nurse-led intervention on dyspnoea severity and psychological well-being in individuals with Chronic Obstructive Pulmonary Disease (COPD). The study encompassed pre-test and post-test evaluations, with an experimental group undergoing the intervention and a control group receiving standard treatment. The study was performed in the Thoracic Ward at Rajiv Gandhi Government General Hospital (RGGGH), Chennai. Sixty individuals diagnosed with COPD were chosen by a non-randomized convenience sample method. Participants were divided into two groups: 30 in the experimental group and 30 in the control group. The inclusion criteria included COPD patients aged 40 to 65 years, who were clinically stable, willing to participate, and able to provide informed permission. Individuals with terminal illnesses, psychological disorders, cognitive impairments, or those engaged in other clinical trials were excluded. The sample size was determined using a previous prevalence of 54% for dyspnoea severity in COPD, with a confidence level of 95% and a relative precision of 33%. Ethical clearance was obtained from the Institutional Ethics Committee (IEC-MMC/NO-44112024), and the hospital granted administrative approval. Informed consent was secured from all participants. Baseline data, including demographic and clinical variables, were collected using a structured questionnaire. The Modified Medical Research Council (mMRC) Dyspnea Scale and a standardized 18-item Psychological Well-Being Scale were used for pre- and post-test evaluations. Data collection was conducted over four weeks. Post-test assessment occurred 21 days after the intervention. The experimental group received a structured 30-minute nurse-led intervention session covering five domains: (1) breathing exercises (pursed-lip and diaphragmatic breathing), (2) light physical activity, (3) dietary guidance tailored for COPD management, (4) psychological support using relaxation techniques, and (5) education on self-management and inhaler technique. Interventions were delivered through demonstration and patient education booklets. The control group received standard medical care without additional intervention.

## Results:

The study included 60 patients with Chronic Obstructive Pulmonary Disease (COPD), evenly distributed into experimental and control groups (n=30 each). In both groups, the majority of participants were above 60 years of age (63.33% experimental; 56.67% control) and male (66.67% experimental; 53.33% control). Most participants had only primary education (23.33% experimental; 20.00% control), were married (26.67% experimental; 30.00% control), and belonged to extended families. Most participants had no smoking habit (26.67% experimental, 30.00%control). The monthly income of most participants ranged from ₹19,759 to ₹26,354 (40.00% experimental; 30.00% control). Chi-square tests showed no significant differences between groups across socio demographic and clinical variables (p>0.05). The severity of dyspnoea was significant in both groups, with 86.67% of the experimental group and 83.33% of the control group indicating severe dyspnoea. Regarding psychological well-being, 56.67% of the experimental group and 63.33% of the control group reported poor levels. No significant differences were seen between the groups at baseline regarding dyspnoea or psychological well-being (p>0.05). Following the nurse-led intervention, a marked improvement was observed in the experimental group. Mild dyspnoea was reported by 56.67%, and none experienced severe dyspnoea, whereas in the control group, 70.00% still had severe dyspnoea. For psychological well-being, 26.67% of the experimental group reached high levels, compared to none in the control group. Statistical analysis showed a significant reduction in dyspnoea scores in the experimental group (mean pre-test: 3.30; post-test: 1.80; p=0.001). Psychological well-being scores improved significantly in the experimental group (mean pre-test: 56.90; post-test: 89.47; p=0.001). No significant changes were observed in the control group. Percentage gain scores indicated a 37.5% improvement in dyspnoea and 25.85% in psychological well-being among the experimental group. Age and residence were significantly associated with post-test dyspnoea levels (p=0.05). Gender, age, and residence also showed a significant association with psychological well-being (p=0.05). No significant associations were noted in the control group.

In Table 1 (see PDF), the effectiveness of the nurse-led intervention on dyspnoea severity is evident through the significant improvement observed in the experimental group. At pretest, the mean severity of dyspnoea score for this group was 3.3, which accounted for 82.5% of the maximum score. After the intervention, the mean score dropped to 1.8, which represents a 45.0% severity level, showing a clear reduction in dyspnoea severity. In contrast, the control group showed only a minor decrease in the severity score, from a mean of 3.23 (80.75% of the maximum score) at pretest to 3.07 (76.75% of the maximum score) at post-test. This demonstrates a small change in severity for the control group compared to the experimental group, highlighting the effectiveness of the intervention. Table 2 (see PDF) demonstrates the positive impact of the nurse-led intervention on psychological well-being, as seen in the experimental group. At pretest, the mean psychological well-being score was 56.9, which represented 45.16% of the maximum score. After the intervention, the score increased to 89.47, which corresponds to 71.01% of the maximum score, showing a notable improvement in psychological well-being. Conversely, the control group experienced only a slight increase in their well-being scores, from 57.17 (45.37% of the maximum score) at pretest to 58.16 (46.15% of the maximum score) at post-test, indicating that the intervention had a much more significant effect on the experimental group compared to the control group.

## Discussion:

This study revealed significant improvements in both dyspnoea severity and psychological well-being subsequent to a nurse-led intervention. The experimental group exhibited a significant decrease in dyspnoea severity, transitioning from primarily severe pre-intervention (86.67%) to largely mild post-intervention (56.67%), with no individuals suffering severe symptoms after the intervention. On the other hand, the control group consistently reported elevated severity, underscoring the efficacy of tailored nursing interventions in mitigating dyspnoea. The noted enhancement in dyspnoea corresponds with a recent study by Rafael Henriques *et al.* (2024) demonstrating that nurse-led interventions, including tailored breathing exercises and patient education, significantly diminish dyspnoea severity in individuals with chronic respiratory ailments [11]. A previous study by Ceyhan *et al.* (2022) also indicated that structured nursing care markedly alleviated dyspnoea and enhanced quality of life, highlighting the advantages of specialised nursing methodologies in respiratory care management [12]. The experimental group exhibited a notable enhancement in psychological well-being, evidenced by elevated scores following the intervention. Approximately 26.67% attained high psychological well-being, in sharp contrast to the control group, where no participants reached this level. This result aligns with previous study by Leyro *et al.* (2021) indicating that therapies targeting respiratory symptoms and patient education concurrently enhance psychological resilience while diminishing anxiety and depression in individuals with respiratory diseases [13]. Similarly, age and residency proved to be significant factors correlated with dyspnoea levels following the intervention, suggesting that demographic aspects may affect treatment responsiveness. Previous research by Joshi (2024) has indicated that older age correlates with increased dyspnoea severity following intervention, implying that customised therapies may be necessary for senior populations [14]. Gender, age and residency were substantially correlated with psychological well-being, corroborating previous study by Aldhahir (2024) which highlight the importance of demographic characteristics in the psychological outcomes of patients with respiratory diseases [15]. Future research should incorporate longitudinal studies to evaluate enduring benefits and possible modifications in intervention tactics according to demographic variations. The nurse-led intervention offers a feasible and efficient method for enhancing both dyspnea severity and psychological well-being in individuals suffering from chronic dyspnoea.

## Conclusion:

The effectiveness of nurse-led interventions in managing COPD, improving both dyspnoea and psychological well-being is shown. The approach includes education, breathing exercises, and emotional support, led to better patient control, adherence and quality of life. Thus, we show the integration of non-pharmacological strategies into routine COPD care, emphasizing the importance of trained nurses in chronic disease management.
